# Enhancing the Antibacterial Activity of Endolysin against *Klebsiella pneumoniae* through Fusion Engineering Using Antimicrobial Peptide Sub5

**DOI:** 10.4014/jmb.2601.01025

**Published:** 2026-06-11

**Authors:** Kexuan Gao, Yuhui Li, Yuxuan Wang, Chuanyan Zheng, Keyu Zhu, Xinru Mao, Jinlong Bai, Ruirui Hu, Bingbing Lei, Shengwei Hu, Xia Li, Wei Ni

**Affiliations:** 1 College of Life Sciences, Shihezi University, Shihezi City, Xinjiang 832003, P. R. China; 2 Xinjiang Academy of Agricultural and Reclamation Science, Shihezi City, Xinjiang 832000, P. R. China; 3 Opthalmic Center, Xinjiang 474 Hospital, Urumqi City, Xinjiang 830013, P. R. China

**Keywords:** *Klebsiella pneumoniae*, Endolysin, Antimicrobial peptide, Antimicrobial activity, Skin infection

## Abstract

The majority of clinically isolated *Klebsiella pneumoniae* (*K. pneumoniae*) strains are opportunistic pathogens exhibiting multidrug resistance (MDR), which has driven the exploration of alternative therapeutic approaches. Endolysins, as phage-derived lytic enzymes, represent highly promising novel antimicrobial agents. However, their application against Gram-negative bacteria is constrained by the physical barrier of the outer membrane (OM). Therefore, conferring the ability for endolysins to penetrate or disrupt the OM, thereby reaching the target peptidoglycan, is crucial. In this study, we fused the antimicrobial peptide (AMP) Sub5 with LysK1, a phage-derived endolysin targeting *K. pneumoniae*. Leveraging Sub5's membrane-targeting mechanism, we enabled LysK1 to traverse the OM barrier of *K. pneumoniae*, targeting the peptidoglycan layer to exert synergistic antimicrobial effects. The results showed that, compared with LysK1, LysK1-Sub5 reduced the minimum inhibitory concentration (MIC) against *K. pneumoniae* by 2.7 to 6.1-fold and was able to inhibit the growth of *K. pneumoniae* within 16 h without the need for an OM permeabilizer. In addition, it exhibited stable environmental tolerance and broad-spectrum lytic activity. *In vivo* experiments using a mouse skin wound infection model demonstrated that LysK1-Sub5 accelerated wound healing and effectively reduced *K. pneumoniae* infection. This indicates LysK1-Sub5’s potential as a topical antimicrobial agent against *K. pneumoniae* infections.

## Introduction

*Klebsiella pneumoniae* is a major opportunistic pathogen in both community and healthcare environments [1]. It commonly colonizes the skin, gastrointestinal tract, and respiratory tract of healthy individuals [2, 3]. In immunocompromised hosts, such as those with weakened immunity, diabetes, or burn injuries, it frequently causes infections, with skin and soft tissue infections, including burn wounds, being especially common [4-6]. These infections can progress to severe invasive conditions such as pneumonia and sepsis [7]. The mortality rate associated with these invasive infections exceeds 40%, representing a serious threat to patient health [4, 8, 9]. In recent years, the emergence of MDR strains, particularly carbapenem-resistant *K. pneumoniae* (CRKP), has posed substantial challenges for clinical treatment [10-12]. Antimicrobial resistance (AMR) poses a major global health threat, directly causing 1.14 million deaths in 2021. *K. pneumoniae* ranks as the second leading cause of AMR-related fatalities. Without intervention, AMR-attributable deaths are projected to reach 1.91 million by 2050, with cumulative deaths exceeding 39 million between 2025 and 2050 [13]. Consequently, the World Health Organization (WHO) has classified CRKP as a critical pathogen requiring strengthened prevention, control, and targeted therapeutic strategies [14]. Infection rates and mortality due to CRKP are notably higher in intensive care units (ICUs) [15, 16]. Moreover, its rapid transmission within ICUs and the emergence of hypervirulent *K. pneumoniae* (hvKP) further complicate infection control efforts [17, 18]. There is thus an urgent need to develop novel antimicrobial agents to combat infections caused by MDR *K. pneumoniae*.

In view of the limitations of traditional control methods, alternative strategies are currently in the active research stage. Among them, the application of endolysin derived from bacteriophage has attracted considerable attention. Endolysin is an enzyme encoded by bacteriophages, which can specifically degrade the bacterial cell wall, thus inducing rapid bacterial lysis [19, 20]. Notably, even when produced as recombinant proteins, these enzymes retain strong bactericidal activity [21]. In Gram-positive bacteria, which lack an OM, endolysins can directly access and cleave the peptidoglycan layer, leading to efficient cell lysis [22, 23]. Consequently, endolysin-based therapeutics targeting Gram-positive pathogens have become a primary research focus. For example, He Liu *et al*. (2024) developed a pH-responsive hydrogel by loading LysSYL onto the self-assembling peptide L5 (denoted as L5@LysSYL), and this system demonstrated efficacy against methicillin-resistant *Staphylococcus Aureus* (*S. Aureus*) [24]. In contrast, Gram-negative bacteria possess an OM that acts as a physical barrier, preventing endolysins from accessing the peptidoglycan layer and thus significantly reducing their lytic activity [25, 26]. To address this limitation, studies exploring endolysins for Gram-negative bacteria often incorporate OM permeabilizers, such as ethylenediaminetetraacetic acid (EDTA) or cell-penetrating peptides, to facilitate endolysin translocation [27]. AMPs represent another promising solution. Due to their unique physicochemical and structural properties, AMPs can interact directly with lipopolysaccharides on the OM of Gram-negative bacteria, forming pores that disrupt bacterial integrity and metabolism [28-30]. This mechanism has inspired the development of “Artilysins”, which are engineered lysins fused with AMPs, as a potent strategy to combat resistant pathogens [31]. For instance, Art-085 (a fusion of the AMP SMAP-29 and the modular Artilysin KZ144, derived from the giant *Pseudomonas* phage phiKZ) exhibits enhanced activity against Gram-negative bacteria, and it does not require EDTA or organic acids for membrane penetration [32]. Its derivative, Art-175, shows improved stability and lytic activity against multiple clinical isolates of *Pseudomonas aeruginosa* (*P. aeruginosa*), *Escherichia coli* (*E. coli*), and *Acinetobacter baumannii* (*A. baumannii*) [33, 34]. Similarly, fusing eight amino acids from the AMP Cecropin A to either the N-terminus or C-terminus of LysAB2 increased the enzyme’s activity against *A. baumannii* by 3-log and >5-log, respectively [35]. These findings highlight the substantial potential of AMP fusion in enhancing the antibacterial efficacy of Artilysins.

The endolysin LysK1 selected for this study is a protein annotated in databases as originating from the *K. pneumoniae* strain IS43, with its sequence information listed in the TrEMBL database. This study experimentally demonstrated for the first time that LysK1 can specifically target and lyse clinical isolates of *K. pneumoniae*; its antibacterial activity was experimentally validated for the first time in this study. In this study, Sub5 was selected as the fusion partner for LysK1. As a short-chain, cationic and amphipathic AMP, Sub5 can bind to bacterial membrane structures through electrostatic interaction, penetrate the membrane barrier into the cytoplasm in a non-endocytic manner, disrupt membrane integrity and interfere with intracellular energy metabolism, ultimately leading to bacterial lysis [36-38]. Moreover, its compact structure reduces interference with the folding and activity of the fused endolysin. Therefore, Sub5 was adopted in this work to enhance the antibacterial activity of LysK1 against *K. pneumoniae*.

In this study, AMP Sub5 was fused with the endolysin LysK1. Using the OM targeting characteristics of Sub5, the fusion protein promotes LysK1 to pass through the OM barrier of *K. pneumoniae*, so that LysK1 can target peptidoglycans and play a synergistic antibacterial role. In addition, the lytic potential of Artilysin LysK1-Sub5 on the *K. pneumoniae* strain was also studied, and the fusion protein was finally applied to the treatment of mouse skin damage infection model. This method represents a relatively novel and promising antibacterial discovery strategy, which is expected to provide a new way to cope with the challenge of antibiotic resistance.

## Materials and Methods

### Plasmids, Bacterial Strains and Growth Conditions for Chemicals, Reagents, and Instruments

The bacterial strains used in this section were sourced as follows: *K. pneumoniae* ATCC 13883 and *S. aureus* ATCC 43300 were obtained from the Shanghai Bioresource Collection Center (SHBCC); *A. baumannii* CMCC 25001, *A. baumannii* ATCC 19606, and *P. aeruginosa* PAO1 were from the Shanghai Microbial Culture Collection (SHMCC); *E. coli* ETEC K88, *E. coli* ETEC K99, *Salmonella typhimurium* (*S. typhimurium*) ATCC 14028, and *Salmonella enterica* (*S. enterica*) ATCC 9150 were acquired from the Beijing BNCC Biotechnology Research Institute (BNCC). The remaining *K. pneumoniae* strains were isolated from bovine samples collected across six provinces in China (Guangzhou, Shandong, Gansu, Hunan, Ningxia, and Sichuan), representing 11 distinct sequence types (STs), and were preserved in our laboratory. The endolysin LysK1 used in this experiment was synthesized by Nanjing Zhongding Biotechnology Co., Ltd. and is preserved in our laboratory. The pCold-SUMO vector and gene used in this experiment were synthesized by Sangon Biotech (China) Co., Ltd. The competent cells for expression were RTS BL21(DE3) Chaperone solubilizing expression competent cells (Catalog No.: ZY-6K0003-10T), which were purchased from Shanghai Zeye Biotechnology Co., Ltd.All bacterial strains were cultured in Luria-Bertani broth (LB) or Mueller-Hinton broth (MHB) (supplemented with 1.5% agar when preparing solid media) at 37°C or 15°C. Ampicillin and chloramphenicol were added to the culture media as required. LB broth and MHB broth were purchased from Beijing Aoboxing Biotechnology Co., Ltd., agar from Anhui Biosharp Biotechnology Co., Ltd., and ampicillin and chloramphenicol from Beijing Solarbio Science & Technology Co., Ltd.

### Docking Domain Prediction, 3D Structure Analysis, and Molecular Docking

We used InterProScan to predict the protein domains of LysK1-Sub5. The three-dimensional structures of LysK1 and LysK1-Sub5 were predicted using AlphaFold3. The peptidoglycan fragment (ligand) was downloaded from PubChem, preprocessed with AutoDock Tools (ADT), and docked to the receptor whose active site was defined by a grid box using AutoDock. The docking results were analyzed with the Analyze module, visualized in PyMOL 3.1.3, and exported as images.

### Cloning, Expression, Purification and Quantification of LysK1-Sub5

The target gene sequence construction for LysK1-Sub5 comprises three components: endolysin LysK1, a linker, and the AMP Sub5 (Table S1). Entrust Sangon Biotech (Shanghai) Co., Ltd. to clone the target gene into the host *E. coli* TOP10 via vector construction by SpeI/XbaI double digestion, with ampicillin resistance as the screening marker. *E. coli* TOP10 harboring the target plasmid was inoculated at a 1:100 ratio into fresh LB medium supplemented with ampicillin (the corresponding antibiotic). The culture was incubated overnight at 37°C with shaking at 200 rpm, and plasmid extraction was conducted on the following day. Subsequently, the extracted plasmid DNA (10–100 ng) was transformed into BL21(DE3) Chaprone competent cells via the heat shock method.

The expression strain BL21(DE3) was inoculated into 20 mL LB medium supplemented with 20 μg/mL ampicillin and 20 μg/mL chloramphenicol, followed by overnight incubation at 37°C with vigorous shaking. On the following day, the activated strain was inoculated at a 1:50 ratio into 300 mL fresh LB medium containing 20 μg/mL ampicillin, 20 μg/mL chloramphenicol, and 0.5 mg/mL L-arabinose. The culture was incubated at 37°C with vigorous shaking until the OD_600_ reached approximately 0.3. Subsequently, tetracycline was added to a final concentration of 2 ng/mL (to induce the expression of the Chaprone chaperone protein), and shaking was continued at 37°C until the OD_600_ reached approximately 0.5. Isopropyl β-D-1-thiogalactopyranoside (IPTG) was then added to a final concentration of 1 mM, and the culture was incubated at 15°C with shaking for 24 h. The induced bacterial culture was centrifuged at 8000 rpm at room temperature for 5 min, and the bacterial cells were collected. Non-denaturing lysis buffer was added to the collected cells, followed by incubation at 4°C overnight for lysis. On the following day, use an ultrasonic cell disruptor (150 W) to crush cells, The disrupted mixture was centrifuged at 12,000 rpm and 4°C for 30 min; the supernatant was collected and filtered through a 0.22 μm membrane.For column purification: A 5 mL HisTrap™ FF crude column was first pre-equilibrated with 5×column volume of Washing Buffer at a flow rate of 1 mL/min. The filtered supernatant was then loaded onto the pre-equilibrated column at a flow rate of 0.5 mL/min. After loading, the column was washed with 20×column volume of Washing Buffer (50 mM NaH_2_PO_4_, 20 mM imidazole, 300 mM NaCl, pH 8.0) at a flow rate of 1 mL/min. Subsequently, the target protein was eluted with 10 × column volume of Elution Buffer (50 mM NaH_2_PO_4_, 250 mM imidazole, 300 mM NaCl, pH 8.0) at the same flow rate, and the eluate was collected. The collected protein solution was transferred to a Millipore ultrafiltration centrifuge tube (15 mL, 10 kDa molecular weight cutoff) for desalting and concentration. The purified protein was then subjected to His-SUMO tag removal, followed bysodium dodecyl sulfate-polyacrylamide gel electrophoresis (SDS-PAGE) to verify the size of the target protein band. The protein concentration was determined using a BCA protein concentration assay kit, and the purified protein was aliquoted and stored at -80°C.

### MICs Assay

The Minimal inhibitory concentrations (MICs) of LysK1 and LysK1-Sub5 were determined via the broth dilution method. The test strains included *K. pneumoniae* ATCC 13883 and 11 clinical isolates of *K. pneumoniae* with ST1537, ST1480, ST7136, ST7139, ST7134, ST5507, ST7138, ST49, ST7130, ST661, and ST2239. First, all bacterial strains were cultured to the logarithmic growth phase, then diluted in MHB to a final concentration of 1 × 10^6^ CFU/mL. For the LysK1 group: the prepared bacterial suspension was treated with EDTA prior to subsequent steps (to facilitate LysK1-mediated OM penetration); no EDTA treatment was required for the LysK1-Sub5 group. Next, 100 μL of the bacterial suspension was added to each well of a microtiter plate. Subsequently, 100 μL of purified LysK1 or LysK1-Sub5 was added to the corresponding wells, followed by two-fold serial dilution of the protein to generate a concentration gradient. Finally, each well (containing 200 μL of the bacterial-protein mixture) was incubated at 37°C for 16 hours. A bacterial suspension without any protein (*i.e.*, 100 μL MHB + 100 μL bacterial suspension) was used as the negative control. The MIC was defined as the lowest concentration of LysK1 or LysK1-Sub5 at which complete inhibition of bacterial growth was visually observed. All experiments were performed in biological triplicates to ensure reproducibility.

### Effects of Temperature, NaCl Concentration and pH on Endolysin Activity

To investigate the effect of temperature on the bactericidal activity of LysK1-Sub5, *K. pneumoniae* ATCC 13883 in the logarithmic growth phase was selected, and the concentration of the bacterial suspension was adjusted to 1 × 10^6^ CFU/mL. Among them, the bacterial suspension for incubation with LysK1 was pretreated with EDTA. LysK1-Sub5 (final concentration: 25.3 μg/mL) and LysK1 (final concentration: 67.9 μg/mL) were pretreated in a water bath at 4, 25, 37, and 45°C for 1 h, respectively, and then added to the corresponding bacterial suspensions. All groups were incubated with shaking at 37°C and 180 rpm for 2 h. Subsequently, the samples were serially diluted, plated on agar plates, and colony-forming units (CFU) were counted. A bacterial mixture without protein was used as the negative control. To investigate the effect of NaCl concentration on the bactericidal activity of LysK1-Sub5, *K. pneumoniae* ATCC 13883 in the logarithmic growth phase was used. The bacterial cells were resuspended three times in PBS buffers containing different NaCl concentrations (50, 100, 200, and 500 mmol/L), and the concentration of the bacterial suspension was adjusted to 1 × 10^6^ CFU/mL. The bacterial suspension for incubation with LysK1 was pretreated with EDTA. LysK1-Sub5 (final concentration: 25.3 μg/mL) and LysK1 (final concentration: 67.9 μg/mL) were respectively added to the bacterial suspensions of each group. All groups were incubated with shaking at 37°C and 180 rpm for 2 h. After incubation, the samples were serially diluted, plated on agar plates, and CFU were counted. A bacterial mixture without protein was used as the negative control. To investigate the effect of pH on the bactericidal activity of LysK1-Sub5, *K. pneumoniae* ATCC 13883 in the logarithmic growth phase was used. The bacterial cells were resuspended three times in PBS buffers with different pH values (4, 6, 7, 8, and 9), and the concentration of the bacterial suspension was adjusted to 1 × 10^6^ CFU/mL. The bacterial suspension for incubation with LysK1 was pretreated with EDTA. LysK1-Sub5 (final concentration: 25.3 μg/mL) and LysK1 (final concentration: 67.9 μg/mL) were respectively added to the bacterial suspensions of each group. All groups were incubated with shaking at 37°C and 180 rpm for 2 h. After incubation, the samples were serially diluted, plated on agar plates, and CFU were counted. A bacterial mixture without protein was used as the negative control.

### Evaluation of the Time-Course Antibacterial Curves of LysK1 and LysK1-Sub5

A single colony of *K. pneumoniae* ATCC 13883 cultured overnight at 37°C on LB agar plates was picked with an inoculating loop and inoculated into MHB broth. After shaking incubation, the bacterial suspension was adjusted to a 0.5 McFarland turbidity standard, then serially diluted to a final concentration of approximately 1 × 10^6^ CFU/mL for subsequent use. For the group treated with LysK1 following EDTA pretreatment, the bacterial suspension was pre-treated with EDTA to a final concentration of 1 mM in the system, mixed thoroughly, and kept still before use; no EDTA pretreatment was applied to the suspensions of the other groups. Each prepared bacterial suspension was fully mixed with the corresponding test reagents, and incubated at a constant temperature of 37°C in an automatic microplate reader. The OD_600_ value was measured every 30 min during incubation to reflect changes in bacterial density, and the experiment was performed in three biological replicates.

The MIC of LysK1 against *K. pneumoniae* ATCC 13883 was determined to be 67.9 μg/mL, and the MIC of LysK1-Sub5 against *K. pneumoniae* ATCC 13883 was 25.3 μg/mL. The test groups included: (1) EDTA-untreated fusion protein LysK1-Sub5 group at concentrations of 1× MIC and 1/2× MIC; (2) EDTA-treated LysK1 group at concentrations of 1× MIC and 1/2× MIC; (3) EDTA-untreated LysK1 group at concentrations of 1× MIC and 1/2× MIC; (4) PBS control group with an equal volume of PBS buffer added only.

### LysK1-Sub5 Lytic Spectrum Evaluation

*A. baumannii* CMCC 25001, *A. baumannii* ATCC 19606, *P. aeruginosa* PAO1, *E. coli* ETEC K88, *E. coli* ETEC K99, *S. Typhimurium* ATCC 14028, *S. Enterica* ATCC 9150 and *S. aureus* ATCC 43300 were cultured to the logarithmic growth phase with an OD_600_ of 0.6. The cultures were centrifuged at 8000 rpm for 5 min at 4°C, and the bacteria were resuspended three times in sterile 1 × PBS buffer (pH = 7.5). The bacterial suspension was adjusted to 1 × 10^6^ CFU/mL for later use. Subsequently, LysK1 and LysK1-Sub5 were added to reach a final concentration of 25.3 μg/mL, with an equal volume of 1 × PBS buffer set as the control. All mixtures were incubated with shaking at 180 rpm and 37°C for 2 h. The samples were serially diluted, plated, and the colonies were counted.

### Therapeutic Effects of LysK1-Sub5 on Skin Injury Infection Models in BALB/c Mice

All animal experiments in this study were strictly conducted in accordance with the relevant guidelines for the welfare and management of laboratory animals issued by the Ministry of Science and Technology of China. The principle of humanitarian care was followed throughout the study to minimize stress and pain in experimental animals. The relevant experimental protocol was reviewed and approved by the Laboratory Animal Ethics Committee of Shihezi University, with the ethical approval number A2025-992. Six-week-old female BALB/c mice were used in the experiments and provided by Hunan SJA Laboratory Animal Co., Ltd. The mice were housed in an Specific Pathogen Free (SPF)-level individually ventilated cage system at the Animal Genetic Engineering Laboratory of Shihezi University. The environmental parameters were controlled as follows: temperature at 23°C ± 3°C, relative humidity of 60% ± 10%, with a 12-hour/12-hour reverse light-dark cycle implemented.

In this study, the tape stripping (TAP) method was adopted to establish a superficial skin wound infection model in mice. Mice were anesthetized with Avodine (a ready-to-use tribromoethanol solution). Subsequent operations were performed once deep anesthesia was achieved. The dorsal hair of the mice was shaved, and the exposed skin was disinfected with 75% alcohol. Sterile medical tape was then repeatedly applied to the skin area until erythema, petechial hemorrhage or blisters appeared on the skin. The mice were reared individually after modeling to prevent scratching and biting between individuals. Based on *in vitro* MIC values and preliminary pilot studies, we applied a topical dose equivalent to the MIC value per wound site.

After successful model establishment, 20 μL of logarithmic-phase *K. pneumoniae* ATCC 13883 suspension (5 × 10^6^ CFU/mL) was inoculated onto the injured skin of each mouse. Two hours post-infection, the mice were randomly divided into three groups (n = 10 per group): the PBS control group (treated with PBS), the LysK1-Sub5*1 group (treated with 25.3 μg of LysK1-Sub5), and the LysK1-Sub5*2 group (treated with 25.3 μg of LysK1-Sub5 initially, followed by an equivalent dose of the protein administered 6 hours after the first dose). Partial mice (n = 5 per group) were euthanised 24 hours after the final administration. The wound tissue was collected, homogenised, subjected to serial dilutions, and aliquots of the diluted tissue homogenate were plated onto MacConkey agar. The agar plates were incubated at 37°C for 18 hours, after which CFUs counts were performed to determine the bacterial load in the wound tissue. During the whole experiment, mice (n = 3 per group) were observed every day. Take photos of the wound and measure the size of the wound on days 0, 2, 4, 6 and 8 after treatment. Use ImageJ software to calculate the wound area and wound healing percentage. In addition, wound tissue samples (n = 2 per group) were collected on the 4th day after treatment, fixed in 4% polyformaldehyde, paraffin encrapment was carried out, stained with haematoxylin and eosin (H&E), and finally histopathological examination was carried out.

### Statistical Analysis

All statistical analyses were performed, and corresponding graphs were generated using GraphPad Prism (Version 10.1.2). Data are presented as the mean normalized to the mean of control values ± standard deviation (SD). Compare the differences in the dependent variable between groups at different levels of a single categorical factor using one-way analysis of variance (ANOVA), and explore the main and interactive effects of two categorical factors on the dependent variable using two-way ANOVA. Statistical significance was defined as follows: *p < 0.05; **p < 0.01; ***p < 0.001; ns, not significant. The graphical abstract is drawn using BioGDP[39].

## Results

### Design of LysK1-Sub5 Fusion Protein Targeting *K. pneumoniae*

**Fig. 1:**
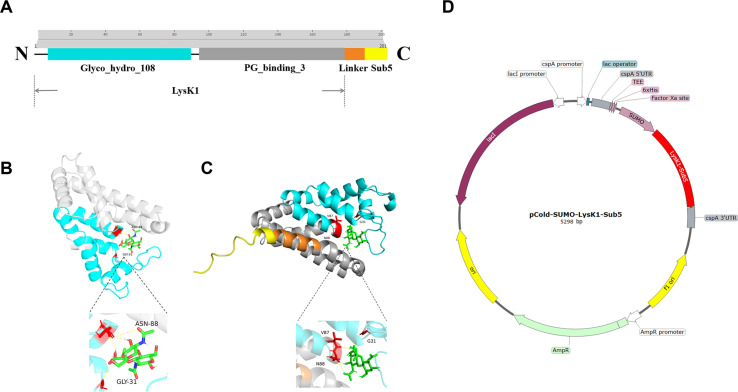
Design of LysK1-Sub5 Fusion Protein Targeting *K. pneumoniae*. (**A**) Domain prediction of LysK1-Sub5. Cyan: Glycosyl hydrolase catalytic domain (Glycosyl_hydrolase_108, PF05838) of endolysin LysK1; Gray: PG_binding_3 (PF09374) cell wall-binding domain; Orange: Linker: EAAAKEAAAK; Yellow: AMP Sub5: RRWKIVVIRWRR. (**B**) Molecular docking diagram of LysK1 with the NAM-NAG fragment. Red: Key amino acid residues of LysK1 interacting with the NAM-NAG fragment; Green: NAM-NAG fragment; Yellow dashed lines: Hydrogen bonds formed in molecular docking. (**C**) Molecular docking diagram of LysK1-Sub5 with the NAM-NAG fragment. Red: Key amino acid residues of LysK1-Sub5 interacting with the NAM-NAG fragment; Green: NAM-NAG fragment; Yellow dashed lines: Hydrogen bonds formed in molecular docking; Orange: Linker; Yellow: AMP Sub5. (**D**) Plasmid construction map of LysK1-Sub5.

Previously, our laboratory validated that endolysin LysK1 exerts targeted lytic activity against *K. pneumoniae* to varying degrees. Sub5 is a cationic peptide consisting of 12 amino acids, which exhibits broad-spectrum antibacterial activity against both bacteria and fungi. To enhance the membrane permeability and antimicrobial efficacy of LysK1, Sub5 was fused to the C-terminus of LysK1 via a rigid linker, yielding the fusion construct designated as LysK1-Sub5 (Table S1). The structural domain of LysK1 is as follows: it contains an N-terminal glycosyl hydrolase catalytic domain (Glycosyl_hydrolase_108, PF05838) and a C-terminal cell wall-binding domain (PG_binding_3, PF09374). The amino acid sequence of the rigid linker is EAAAKEAAAK, and the amino acid sequence of AMP Sub5 is RRWKIVVIRWRR (Fig. 1A). In this study, molecular docking simulations were performed for LysK1, LysK1-Sub5 with the peptidoglycan N-Acetylmuramic Acid-N-Acetylglucosamine (NAM-NAG) fragment, to predict their targeted binding characteristics to the bacterial cell wall at the bioinformatics level. The simulation results showed that both LysK1 and LysK1-Sub5 had the potential to target and bind to the bacterial cell wall, and could form hydrogen bonds (yellow dashed lines) at the active sites of NAM-NAG. The shared binding sites of the two proteins were located at the 31st and 88th amino acids, with glycine at the 31st residue and asparagine at the 88th residue (Fig. 1B and 1C). On this basis, bioinformatics prediction indicated that after fusion with the AMP Sub5, LysK1-Sub5 could additionally bind to the valine residue at position 87 (Fig. 1C). These results are only theoretical inferences from simulations, and the relevant binding advantages need to be further verified by subsequent *in vitro* functional experiments. Next, through the SpeI/XbaI double enzyme digestion and directed cloning technology, the target gene fragment with a length of 618 bp was successfully inserted into the multicloning site of the 4.68 kb pCold-SUMO vector, resulting in the construction of the pCold-SUMO-LysK1-Sub5 plasmid containing the target sequence (Fig. 1D). Agarose gel electrophoresis was also performed to verify whether it conformed to the target size (Fig. S1). After purification of the recombinant protein and subsequent removal of the His-SUMO tag, SDS-PAGE analysis was performed to verify the purity and molecular weight of the target protein. The results confirmed that the purified LysK1-Sub5 had a relative molecular mass of 22.6 kDa, consistent with the expected size (Fig. S2). The purified protein was then aliquoted and stored at -80°C for use in subsequent experimental assays.

### LysK1-Sub5 Exhibited a Lower MIC than LysK1

We hypothesized that fusion with Sub5 may enhance the permeability of the bacterial OM, thereby improving the antimicrobial activity of LysK1. To verify this hypothesis, the broth dilution method was employed to determine the antimicrobial activity of LysK1 and LysK1-Sub5 against 12 *K. pneumoniae* strains, including 1 standard strain and 11 bovine isolates. Compared with its parents, LysK1-Sub5 showed a 2.7-fold decrease in MIC against *K. pneumoniae* ATCC 13883, a 4-fold decrease in MIC against *K. pneumoniae* ST7139 and ST49, a 4.6-fold decrease in MIC against *K. pneumoniae* ST1537, a 5.4-fold decrease in MIC against *K. pneumoniae* ST1480, ST7134, ST7130, and ST2239, and a 6.1-fold decrease in MIC against *K. pneumoniae* ST7136, ST5507, ST7138, and ST661. Overall, the MIC range of LysK1-Sub5 against the tested *K. pneumoniae* strains was 22.1 to 25.3 μg/mL, while the MIC range of LysK1 against the tested *K. pneumoniae* strains was 67.9 to 135.8 μg/mL. This confirms that LysK1-Sub5 possesses enhanced antimicrobial activity compared to the parent LysK1 (Table 1).

### Effects of Temperature, NaCl Concentration, and pH on the Bactericidal Activity of LysK1-Sub5

**Fig. 2:**
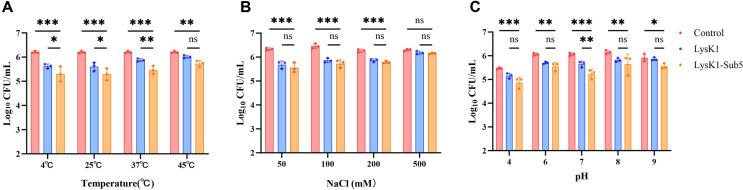
Effects of temperature, NaCl concentration and pH on the lysis of *K. pneumoniae* ATCC 13883 by LysK1-Sub5. (**A**) Effect of temperature on the stability of LysK1 and LysK1-Sub5 after treatment at 4–45°C. (**B**) Effect of NaCl concentration on the stability of LysK1 and LysK1-Sub5 in the range of 50–500 mM. (**C**) Effect of pH on the stability of LysK1 and LysK1-Sub5 after treatment at pH 4–9. Data are presented as Mean ± SD. Error bars represent SD. Statistical significance was assessed by two-way ANOVA. *P < 0.05, **P < 0.01, ***P < 0.001, and ns indicates no significant difference.

The bactericidal activity of LysK1-Sub5 was tested under different temperatures, NaCl concentrations and pH values, and finally evaluated by the plate counting method (Fig. 2). Temperature stability analysis showed that after treatment at 4°C, 25°C, 37°C and 45°C for 1 h, LysK1-Sub5 maintained high bactericidal activity at 4–45°C. At 4–37°C, its activity was significantly increased compared with LysK1. Bactericidal activity assays at NaCl concentrations of 50–500 mM revealed that LysK1-Sub5 exhibited high activity at 50–200 mM NaCl, but showed no significant inhibitory effect at 500 mM NaCl. There was no significant difference in NaCl stability between LysK1-Sub5 and LysK1. Finally, bactericidal activity tests at pH 4, 6, 7, 8 and 9 indicated that LysK1-Sub5 maintained favorable inhibitory activity at pH 4, 6, 7 and 8, but its inhibitory effect was greatly reduced at pH 9. The optimal pH for bactericidal activity of LysK1-Sub5 was 7, and its activity was significantly improved compared with LysK1.

### Time-Course Antibacterial Curve of LysK1 and LysK1-Sub5

**Fig. 3:**
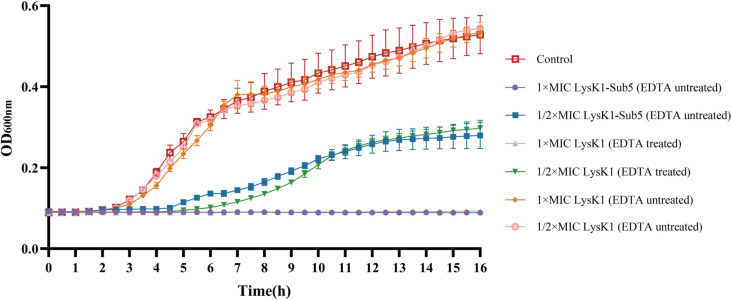
Time-Course Antibacterial Curve of LysK1 and LysK1-Sub5. Data are presented as Mean ± SD. Error bars represent SD.

In this study, the time-course antibacterial curves of LysK1-Sub5 and LysK1 against *K. pneumoniae* ATCC 13883 were analyzed via dynamic monitoring of OD_600_ over a 16-h period. The results showed that the control group, the 1 × MIC LysK1 (EDTA-untreated) group, and the 1/2 × MIC LysK1 (EDTA-untreated) group all entered the rapid growth phase at approximately 2.5 h, and continued to grow steadily within 16 h. In contrast, the OD_600_ values of the 1× MIC LysK1-Sub5 (EDTA-untreated) group and the 1 × MIC LysK1 (EDTA-treated) group remained at their initial levels throughout the 16-hour monitoring period of the time-course antibacterial curve. This directly demonstrated that both the 1 × MIC concentration of LysK1-Sub5 and the 1× MIC concentration of LysK1 could completely inhibit the growth of *K. pneumoniae* ATCC 13883. Meanwhile, it indicated that at the same concentration, LysK1-Sub5 could exert effective antibacterial effects without EDTA pretreatment. For the 1/2 × MIC concentration groups, both the LysK1-Sub5 (EDTA-untreated) group and the LysK1 (EDTA-treated) group maintained stable OD_600_ values during the first 4 h of the time-course antibacterial curve monitoring. Bacterial growth resumed slowly after 4 h, but both the growth rate and final bacterial density were significantly lower than those in the PBS control group. This suggested that the 1/2 × MIC concentration of LysK1-Sub5 and LysK1 could effectively inhibit bacterial growth in the short term, but failed to completely prevent their subsequent regrowth. In summary, LysK1-Sub5 exhibited a concentration-dependent growth inhibitory effect against *K. pneumoniae* ATCC 13883 without EDTA pretreatment of the bacteria, as shown in the time-course antibacterial curves; that is, the higher the concentration of LysK1-Sub5, the stronger the inhibitory activity (Fig. 3).

### The fusion of Sub5 broadened the lytic spectrum of LysK1.

The lytic spectra of LysK1-Sub5 and LysK1 were determined using eight standard strains, including seven Gram-negative bacteria and one Gram-positive bacterium. The seven Gram-negative strains were *A. baumannii* CMCC 25001, *A. baumannii* ATCC 19606, *P. aeruginosa* PAO1, *E. coli* ETEC K88, *E. coli* ETEC K99, *S. Typhimurium* ATCC 14028, and *S. enterica* ATCC 9150. The single Gram-positive strain was *S. aureus* ATCC 43300. The lytic spectrum results showed that the fusion with Sub5 expanded the lytic range of LysK1. LysK1 alone exhibited moderate lytic activity only against *S. enterica* ATCC 9150 and *E. coli* ETEC K99. In contrast, LysK1-Sub5 not only retained moderate lytic activity against *P. aeruginosa* PAO1, *S. enterica* ATCC 9150, and *E. coli* ETEC K99, but also displayed strong lytic activity against *S. Typhimurium* ATCC 14028 and *S. aureus* ATCC 43300. Compared with the PBS control group, the bacterial number was reduced by 1-2 log units (Table 2).

### *In Vivo* Experiment of LysK1-Sub5 Treatment in a Mouse Skin Injury Infection Model

To evaluate the *in vivo* therapeutic efficacy of LysK1-Sub5, experiments were conducted using a BALB/c mouse model of skin injury-associated infection. First, the dorsal skin of BALB/c mice was shaved and disinfected. The superficial layer of the skin was then stripped using adhesive tape until characteristic signs (erythema, pinpoint haemorrhages, and blisters) were observed, to establish the skin injury model. Next, *K. pneumoniae* ATCC 13883 (10^5^ CFU) was inoculated onto the injured skin of all mice. After a 2-h colonisation period to allow bacterial adherence and initial proliferation, the mice with infected skin injuries were randomly divided into three groups and treated with distinct regimens, respectively: (1) LysK1-Sub5*1 group: a single dose of LysK1-Sub5 (25.3 μg); (2) LysK1-Sub5*2 group: a double dose of LysK1-Sub5 (50.6 μg total, with the second 25.3 μg administered 6 hours after the initial dose); (3) PBS control group: an equivalent volume of PBS buffer, which served as the negative control.

**Fig. 4:**
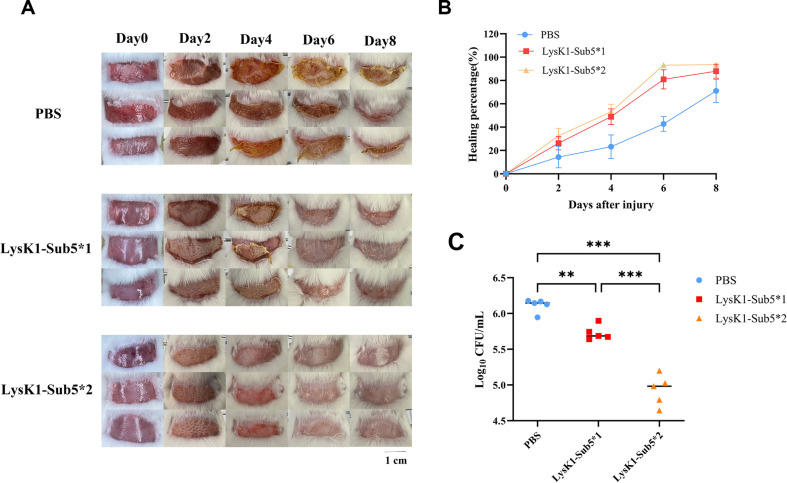
*In vivo* efficacy of LysK1-Sub5 in treating skin wound infections in mice. (**A**) Shows skin wound infection models in mice infected with *K. pneumoniae* ATCC 13883, treated with LysK1-Sub5*1 (25.3 μg), LysK1-Sub5*2 (50.6 μg) and PBS control over 0–8 days. (n = 3 per group) (**B**) Percentage of skin healing in treatment and PBS control groups at 0–8 days. (**C**) Skin wound bacterial load in treatment and control groups at 24 hours (n = 5 per group). Data presented as mean ± SD, error bars denote SD. Statistical significance was assessed by one-way ANOVA. **P < 0.01, ***P < 0.001.

**Fig. 5:**
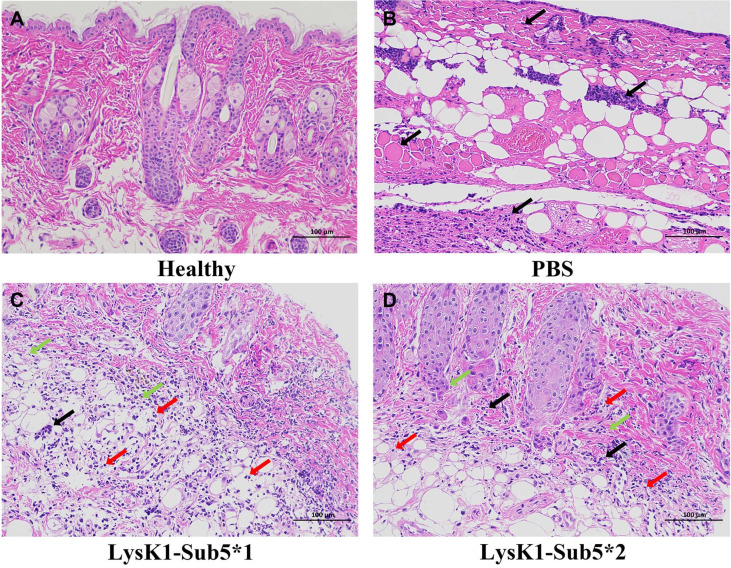
Histological evaluation of mouse skin tissue was conducted using H&E staining (n = 2 per group), with an original magnification of 200×. (**A**) Healthy group. (**B**) PBS group. Black arrow, necrosis. (**C**) treatment group treated with LysK1-Sub5*1. Black arrow, necrotic areas; Red arrow, inflammatory cells; Green arrow, granulation tissue. (**D**) treatment group treated with LysK1-Sub5*2. Black arrow, necrotic areas; Red arrow, inflammatory cells; Green arrow, granulation tissue.

Eight days of continuous observation revealed that wound healing was significantly accelerated in both the LysK1-Sub5*1 group and the LysK1-Sub5*2 group compared to the PBS control group (Fig. 4A). By day 8, the wound healing rate of the PBS group was only 71.04%, while the wound healing rate of the LysK1-Sub5*1 group reached 87.95%, and the wound healing rate of the LysK1-Sub5*2 group reached 93.51% (Fig. 4B). Furthermore, a significant reduction in the skin bacterial load was observed in LysK1-Sub5-treated mice. Specifically, the PBS control group exhibited a skin bacterial load of 1.32 × 10^6^ CFU/mL; in contrast, after treatment with the LysK1-Sub5*1 group, the bacterial load decreased to 5.47 × 10^5^ CFU/mL, corresponding to a 0.39 log unit reduction relative to the PBS group. For the LysK1-Sub5*2 group, the bacterial load was further reduced to 9.32 × 10^4^ CFU/mL, representing a 1.19 log unit reduction compared to the PBS group (Fig. 4C). Concurrently, skin samples were collected from the three treatment groups and a healthy mouse group (serving as the normal control) for histopathological analysis four days post-treatment. Results revealed that the healthy group exhibited intact epidermis with normal morphological structures of skin appendages, including hair follicles and sebaceous glands, with no observable necrosis or other abnormalities (Fig. 5A). In contrast, the PBS group exhibited extensive necrosis extending from the dermis to the subcutaneous layer (black arrows), accompanied by blurred tissue architecture, widespread amorphous eosinophilic material, and abundant necrotic cell debris (Fig. 5B). In mice treated with LysK1-Sub5*1, although areas of necrosis persisted from the dermis to the subcutaneous layer (black arrow), accompanied by increased inflammatory cell infiltration including lymphocytes and granulocytes (red arrow), a small amount of granulation tissue was already present (green arrow) (Fig. 5C). In mice treated with LysK1-Sub5*2, multiple foci of necrosis persisted from the dermis to the subcutaneous tissue (black arrows), yet inflammatory cell infiltration was comparatively reduced (red arrows), with more pronounced granulation tissue formation (green arrows) (Fig. 5D). These histopathological findings further confirm the therapeutic efficacy of LysK1-Sub5 in promoting wound tissue healing in infected mice.

These findings indicate that in a mouse skin injury infection model, LysK1-Sub5 exhibits *in vivo* efficacy in reducing *K. pneumoniae* infection and accelerating wound healing. Consequently, further exploration and optimisation of its therapeutic effects are warranted to develop LysK1-Sub5 as a treatment option for drug-resistant bacterial infections in humans or poultry.

## Discussion

The emergence of MDR strains presents significant challenges for the clinical treatment of *K. pneumoniae* infections. Phage-derived endolysins have emerged as a promising alternative for developing novel antimicrobial agents, owing to their distinct mechanism of action. Although phages typically exhibit a highly specific host range, their corresponding endolysins display a broader lytic spectrum. Specifically, endolysins not only act against more strains within the same bacterial species but also exert lytic activity against bacteria from different genera. For example, LysPBC2 is a natural endolysin derived from *Bacillus cereus* bacteriophages, which has been shown to lyse bacteria belonging to the genera *Bacillus*, *Listeria*, and *Clostridium* [40]. However, Gram-negative bacteria possess a unique OM structure that acts as a physical barrier, preventing endolysins from penetrating to reach and degrade the cell wall peptidoglycan. To address this limitation, endolysins are often combined with OM permeabilisers (*e.g.*, EDTA) when targeting Gram-negative pathogens. For instance, the antimicrobial efficacy of LysG24 and LysCA against *K. pneumoniae* is significantly enhanced when co-administered with EDTA[41]; similarly, LysXFII exhibits no lytic activity against *K. pneumoniae* when used alone, but gains bacteriostatic activity only upon combination with EDTA[42]. Another strategy involves fusing endolysins with different AMPs to generate “Artilysins”. This design leverages the ability of AMPs to penetrate the OM of Gram-negative bacteria [31]. This permeation enables the endolysin moiety to access and degrade the cell wall peptidoglycan, ultimately leading to bacterial lysis. For example, the fusion protein CecA::ST01, formed by combining the endolysin ST01 with the AMP Cecropin A, can effectively eliminate *K. pneumoniae* [43]; another study showed that the fusion proteins ApoE23-LysKp84B and COG133-LysKp84B (generated by fusing the lysin LysKp84B with the AMPs ApoE23 or COG133, respectively) exhibited strong activity, with 10 μM of ApoE23-LysKp84B reducing the CFU of *K. pneumoniae* by more than three orders of magnitude within one hour [44]. Based on the above research results, we attempted to fuse the antibacterial peptide Sub5, which has broad-spectrum antibacterial effects on bacteria and fungi, into the C-terminus of the endostatin LysK1, which has targeted lytic activity against *K. pneumoniae*, and studied its *in vitro* and *in vivo* antibacterial activities.

After completing the sequence design of the fusion protein, molecular docking simulations were performed on the parental LysK1, LysK1-Sub5 and the peptidoglycan monomer NAM-NAG fragment, to predict the targeted binding potential of the two proteins to the bacterial cell wall at the bioinformatics level only. The simulation results showed that both LysK1 and LysK1-Sub5 could bind to glycine and asparagine residues in peptidoglycan (Fig. 1B and 1C). Compared with the parental protein, LysK1-Sub5 could additionally bind to valine residues (Fig. 1C). These predicted results preliminarily indicated that the targeted binding potential of LysK1-Sub5 to NAM-NAG was theoretically better than that of the original LysK1. Meanwhile, model analysis suggested that the rigid linker EAAAKEAAAK could effectively separate the three-dimensional structures of LysK1 and the AMP Sub5 in space, and would not shield the active center sites of LysK1 in theory. This conclusion still needs further verification by subsequent *in vitro* activity experiments.

The antibacterial activity against different clinical isolates of *K. pneumoniae* showed that the MICs of LysK1-Sub5 were 2.7 to 6.1-fold lower than that of the parental LysK1. Broth microdilution tests revealed that the MICs of LysK1 against the tested strains ranged from 67.9 to 135.8 μg/mL, while LysK1-Sub5 inhibited the growth of most strains at only 22.1 to 25.3 μg/mL, with no OM permeabilizer required (Table 1). Consistent with previous reports, this study confirmed that fusion modification with the AMP Sub5 could significantly enhance the antibacterial activity of endolysin against Gram-negative bacteria. The OM barrier of Gram-negative bacteria is a key factor limiting the function of native endolysins. After fusing the membrane-penetrating peptide Sub5 with LysK1, the endolysin could cross the bacterial OM and efficiently target peptidoglycan sites. Islam *et al*. fused Cecropin A to the N-terminus of endolysin, resulting in a 2 to 8-fold reduction in the MICs of the fusion protein, with enhanced activity attributed to OM permeabilization [45].

Subsequently, we inferred the effects of temperature, NaCl concentration and pH on LysK1-Sub5. LysK1-Sub5 maintained high bactericidal activity at 4–45°C, and its activity was superior to that of the parental LysK1 at 4–37°C. LysK1-Sub5 showed strong antibacterial activity at low NaCl concentrations (50–200 mM), while no significant inhibitory activity was observed at 500 mM NaCl. It maintained favorable antibacterial activity at pH 4–8, with the optimal working pH at 7, at which its bactericidal activity was superior to that of parental LysK1 (Fig. 2). Studies have shown that eAbEndolysin, constructed by fusing Cecropin A to the N-terminus of AbEndolysin, exhibits significantly improved stability compared with the parental endolysin, indicating that AMP fusion effectively enhances the stability of endolysins in complex environments[45].

The time-course antibacterial curves showed that LysK1-Sub5 could effectively inhibit bacterial growth within 16 h even in the absence of EDTA, further verifying the mechanism by which Sub5 fusion targets the OM lysis of *K. pneumoniae* (Fig. 3). The antibacterial effect was concentration-dependent, allowing the dosage to be adjusted accordingly in practical applications. This feature is universal. Jeong *et al*. reported that fusing Cecropin A with three endolysins from *E. coli* phages conferred concentration-dependent bactericidal activity against various Gram-negative bacteria[46].

Meanwhile, the fusion of Sub5 broadened the lytic spectrum of LysK1. LysK1 alone showed moderate lytic activity only against *S. enterica* ATCC 9150 and *E. coli* ETEC K99. In contrast, LysK1-Sub5 not only maintained moderate activity against *P. aeruginosa* PAO1, *S. enterica* ATCC 9150 and *E. coli* ETEC K99, but also exerted strong lytic effects on *S. Typhimurium* ATCC 14028 and *S. aureus* ATCC 43300, reducing the bacterial count by 1 to 2 log units compared with the PBS control group (Table 2). In future research, concentration gradient experiments will be added to further quantitatively verify its broad-spectrum antibacterial effect. Studies have shown that the LysC02-AMPs fusion protein significantly broadens the antimicrobial spectrum and exhibits stronger bactericidal activity against various Gram-negative bacteria [47].

Finally, during *in vivo* experiments where LysK1-Sub5 was used to treat *K. pneumoniae*-induced skin wound infections in mice, the LysK1-Sub5*1 and LysK1-Sub5*2 groups showed no excessive scab thickening or secondary infection throughout the trial period—contrasting with the PBS control group. Instead, these treatment groups exhibited faster scab formation and superior wound healing outcomes (Fig. 4A and 4B). Twenty-four hours after protein administration, the skin bacterial load in the LysK1-Sub5*1 group was reduced by 0.39 log units compared to the PBS control group, while the LysK1-Sub5*2 group showed a more pronounced reduction of 1.19 log units (Fig. 4C). These results confirm that LysK1-Sub5 is effective in eradicating *K. pneumoniae* from infected wounds, with higher doses yielding superior therapeutic effects. Histological sections further supported this conclusion (Fig. 5). It should be noted that samples were collected 24 h after protein treatment, which largely eliminated the interference of residual protein on colony counting and ensured more accurate and objective experimental results. In addition, relevant studies have found that the endolysin PlyKp104 can effectively treat skin lesions infected by *K. pneumoniae*[48]. Looking forward, subsequent work may focus on optimising drug delivery formulations (*e.g.*, gels, creams) to enhance LysK1-Sub5’s skin penetration capacity and prolong its retention time in skin tissue. However, it remains unclear whether such formulation optimisations can further improve its therapeutic efficacy against *K. pneumoniae* skin infections.

In conclusion, we successfully engineered an Artilysins LysK1-Sub5 by incorporating the AMP Sub5 into the endolysin LysK1. This fusion protein exhibited potent bactericidal activity in both *in vitro* and *in vivo* assays, and notably, this activity was achieved without the need for OM permeabilisers agents or further protein modifications. This study has certain limitations. The precise bactericidal mechanism of LysK1-Sub5 remains incompletely elucidated, and this therapeutic strategy is still in the preclinical development phase. Although we have presented the current findings as objectively and rigorously as possible, further validation is needed regarding its synergistic antibacterial activity with various antibiotics, its efficacy in eradicating bacterial biofilms, and systematic evaluation in additional *in vivo* infection models. Overall, our study on LysK1-Sub5 provides valuable insights into the emerging field of endolysin-based antimicrobial therapies. It not only enriches the current understanding of fusion endolysin design but also offers an effective strategy and pathway for developing novel countermeasures against infections caused by MDR bacterial strains.

## Supplemental Materials

Supplementary data for this paper are available on-line only at http://jmb.or.kr.



## Figures and Tables

**Table 1:** MICs of LysK1 and LysK1-Sub5 against different *K. pneumoniae* strains.

Strain	Reference or source	MIC (μg/mL)	
		LysK1	LysK1-Sub5
*K. pneumoniae* ATCC13883	SHBCC	67.9	25.3
*K. pneumoniae* ST1537	Laboratory stock	101.9	22.1
*K. pneumoniae* ST1480	Laboratory stock	135.8	25.3
*K. pneumoniae* ST7136	Laboratory stock	135.8	22.1
*K. pneumoniae* ST7139	Laboratory stock	101.9	25.3
*K. pneumoniae* ST7134	Laboratory stock	135.8	25.3
*K. pneumoniae* ST5507	Laboratory stock	135.8	22.1
*K. pneumoniae* ST7138	Laboratory stock	135.8	22.1
*K. pneumoniae* ST49	Laboratory stock	101.9	25.3
*K. pneumoniae* ST7130	Laboratory stock	135.8	25.3
*K. pneumoniae* ST661	Laboratory stock	135.8	22.1
*K. pneumoniae* ST2239	Laboratory stock	135.8	25.3

**Table 2:** The lysis spectra of LysK1 and LysK1-Sub5.

Strain	Reference or source	LysK1	LysK1-Sub5
*P. aeruginosa* PAO1	SHMCC	-	+
*S. typhimurium* ATCC 14028	BNCC	-	++
*S. enterica* ATCC 9150	BNCC	+	+
*E. coli* ETEC K88	BNCC	-	-
*E. coli* ETEC K99	BNCC	+	+
*A. baumannii* CMCC 25001	SHMCC	-	-
*A. baumannii* ATCC 19606	SHMCC	-	-
*S. aureus* ATCC 43300	SHBCC	-	++

Note: “++”: The number of viable bacteria decreased by 1-2, indicating strong lytic activity; “+”: The number of viable bacteria decreased by 0.5-1, and the lytic activity was moderate; “-”: The number of viable bacteria decreased by less than 0.5, with no significant lytic activity.
