# Supplementary material for: Enhancing the Antibacterial Activity of Endolysin against Klebsiella pneumoniae through Fusion Engineering Using Antimicrobial Peptide Sub5
Source: J Microbiol Biotechnol. 2026 Jun 11;36:e2601025. doi: 10.4014/jmb.2601.01025 (PMC13268922; doi:10.4014/jmb.2601.01025)
Supplement: Supplementary file 1 [file jmb-36-e2601025-supple.pdf]

J. Microbiol. Biotechnol. 2026. 36: e2601025

https://doi.org/10.4014/jmb.2601.01025

Supplementary Table and Figures

Name	Amino acid Sequence
LysK1[a]	MTADQIIEGILGKEGGYVDHPSDKGGPTRWGITQTTARAHGYTGD MRNLPRETAKQILLSDYWTGPRFDQVAALSTLLADELCDTGVNMG PSVASKFFQRWLTAMNMRGKLYPDLIPDGAIGPRTITALKGYLSAR GKEGEQVLLRALNCSQGARYLELAEGREANEDFLYGWVKERVL
Linker	EAAAKEAAAK
Sub5	RRWKIVVIRWRR
LysK1-Sub5	MTADQIIEGILGKEGGYVDHPSDKGGPTRWGITQTTARAHGYTGD MRNLPRETAKQILLSDYWTGPRFDQVAALSTLLADELCDTGVNMG PSVASKFFQRWLTAMNMRGKLYPDLIPDGAIGPRTITALKGYLSAR GKEGEQVLLRALNCSQGARYLELAEGREANEDFLYGWVKERVLE AAAKEAAAKRRWKIVVIRWRR

Table S1. Amino acid sequences of LysK1, Linker, Sub5, LysK1-Sub5.

a, The primary accession for LysK1 is W1DTS6.

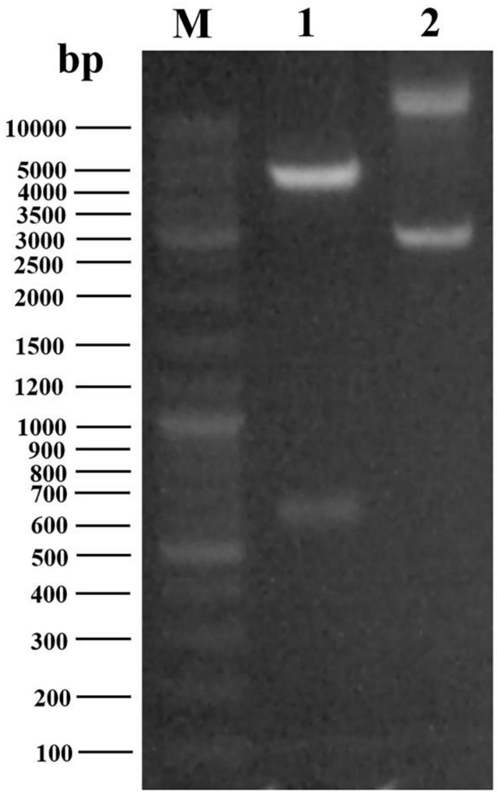

Fig. S1. The results of double enzyme digestion identification of recombinant plasmids were analyzed by agarose gel electrophoresis. Lane M: Marker. Lane 1: The product of SpeI/XbaI double enzyme digestion consists of a 4.68kb vector plasmid fragment and a 618bp target sequence fragment. Lane 2: Original plasmid

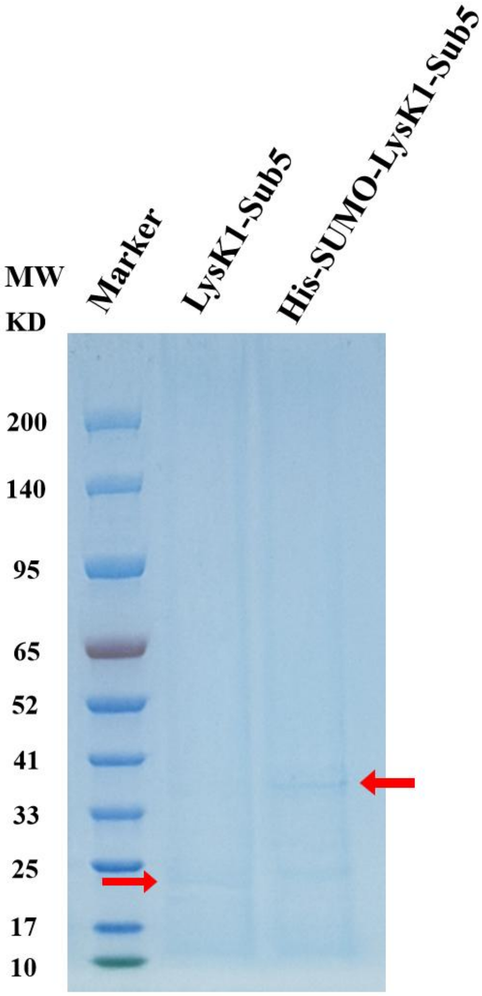

Fig. S2. LysK1-Sub5 protein purification protocol. purified fusion protein separated by SDS-PAGE. Lane 1: Marker. Lane 2: 22.6 kDa LysK1-Sub5. Lane 3: 36.7 kDa His-SUMO-LysK1-Sub5.

Supplementary document 1. Approval Letter of Biology Ethic Committee of Shihezi University.

石河子大学生物伦理委员会伦理审查同意函

Approval Letter of Biology Ethics Committee of Shihezi University

批件号<br>Approval Number	A2025-992			
项目名称<br>Project name	通过融合工程利用抗菌肽Sub5增强内溶素对肺炎克雷伯菌的抗菌活性（Enhancing the Antibacterial Activity of Endolysin Against *Klebsiella pneumoniae* Through Fusion Engineering Using Antimicrobial Peptide Sub5）			
项目来源<br>Sponsor	兵团科技计划、2024 年兵团研究生创新项目等			
研究单位<br>Research department	石河子大学生命科学学院			
主要研究者<br>Main researchers	高柯轩等			
审批材料清单<br>List of Documents				
拟用动物情况<br>Decodes of the animals	动物类别/品系<br>Species of strain	等级<br>Grade	使用数量（♂/♀）<br>Number	动物来源<br>Animal sources
	BALB/c 小鼠	SPF	60	湖南斯莱克景达实验动物有限公司
实验要点<br>Outline of experiments	研究过程中将利用 BALB/c 小鼠开展相关动物实验。利用小鼠皮肤损伤感染模型测试融合蛋白 LysK1-Sub5 的治疗效果，从而评价 LysK1-Sub5 的活性、安全性，以及后续是否能够推广应用。本项目所有实验动物操作严格遵守《实验动物福利与伦理审查指南》、《实验动物饲养管理和使用指南》，并按照石河子大学实验动物伦理委员会制定的规章执行。所有实验动物使用经石河子大学实验动物伦理委员会批准。			
审查意见<br>Evaluation Comments	**Approval 同 意**			
主任委员签字<br>Signature of Chairman	张辉之印			
伦理委员会<br>Ethics Committee	石河子大学生物伦理委员会（盖章）			
日期<br>Date	2025 年 7 月			
